# Activation of Sigma-1 Receptor Alleviates ER-Associated Cell Death and Microglia Activation in Traumatically Injured Mice

**DOI:** 10.3390/jcm11092348

**Published:** 2022-04-22

**Authors:** Mingming Shi, Liang Liu, Xiaobin Min, Liang Mi, Yan Chai, Fanglian Chen, Jianhao Wang, Shuyuan Yue, Jianning Zhang, Quanjun Deng, Xin Chen

**Affiliations:** 1 Department of Neurosurgery, Tianjin Medical University General Hospital, Tianjin 300052, China; mingmingshi96369@hotmail.com (M.S.); ll15588566295@163.com (L.L.); ml970218@163.com (L.M.); wangjianhaosky@163.com (J.W.); yueshuyuan@163.com (S.Y.); jianningzhang@hotmail.com (J.Z.); 2 Tianjin Key Laboratory of Post-Trauma Neuro-Repair and Regeneration in Central Nervous System, Tianjin 300052, China; chaiyankelly@163.com (Y.C.); chenfanglian1976@163.com (F.C.); 3 Tianjin Key Laboratory of Injuries, Variations and Regeneration of Nervous System, Tianjin Neurological Institute, Ministry of Education, Tianjin 300052, China; 4 Baodi Clinical College, Tianjin Medical University, Tianjin 300052, China; mrj20110125@163.com

**Keywords:** traumatic brain injury, endoplasmic reticulum stress, apoptosis, pyroptosis, microglia activation, cerebrovascular function, sigma-1 receptor, PRE-084, BD-1047

## Abstract

Background: Endoplasmic reticulum (ER) stress and unfolded protein response (UPR) is associated with neuroinflammation and subsequent cell death following traumatic brain injury (TBI). The sigma-1 receptor (Sig-1R) acts as a dynamic pluripotent modulator of fundamental cellular processes at the mitochondria-associated membranes (MAMs). The activation of Sig-1R is neuroprotective in a variety of central nervous system diseases, but its impact on ER stress induced by traumatic brain injury is not known. This study investigated the role of Sig-1R in regulating the ER stress-mediated microglial activation and programmed cell death (apoptosis and pyroptosis) induced by TBI. Methods: Ten human brain tissues were obtained from The Tianjin Medical University General Hospital. Four normal brain tissues were obtained from patients who underwent surgery for cerebral vascular malformation, through which peripheral brain tissues were isolated. Six severe TBI tissues were from patients with brain injury caused by accidents. None of the patients had any other known neurological disorders. Mice with Sig-1R deletion using CRISPR technology were subjected to controlled cortical impact-induced injury. In parallel, wild type C57BL/6J mice were analyzed for outcomes after they were exposed to TBI and received the Sig-1R agonist PRE-084 (10 mg/kg daily for three days) either alone or in combination with the Sig-1R antagonist BD-1047 (10 mg/kg). Results: The expression of Sig-1R and the 78 kDa glucose-regulated protein, a known UPR marker, were significantly elevated in the injured cerebral tissues from TBI patients and mice subjected to TBI. PRE-084 improved neurological function, restored the cerebral cortical perfusion, and ameliorated and brain edema in C57BL/6J mice subjected to TBI by reducing endoplasmic reticulum stress-mediated apoptosis, pyroptosis, and microglia activation. The effect of PRE-084 was abolished in mice receiving Sig-1R antagonist BD-1047. Conclusions: ER stress and UPR were upregulated in TBI patients and mice subjected to TBI. Sig-1R activation by the exogenous activator PRE-084 attenuated microglial cells activation, reduced ER stress-associated programmed cell death, and restored cerebrovascular and neurological function in TBI mice.

## 1. Introduction

Traumatic brain injury (TBI) is a major cause of mortality and long-term disability worldwide and a heavy burden to the economy [1]. Secondary brain injury occurs in hours and days following TBI, involving complex and interrelated pathologies that include cellular apoptosis [2], glutamate excitotoxicity [3], ferroptosis [4], pyroptosis [5], and neuroinflammation [6]. Extensive basic and clinical research on TBI in the past has not been translated into successful pharmacological interventions [7].

Secondary cerebral injuries resulting in persistent ER stress have been increasingly recognized for contributing to neuroinflammation and uncontrolled cell death [8,9]. Three signaling pathways of unfolded protein response (UPR) are extensively reported to regulate inflammatory and cell death signaling [10,11,12].

Mitochondria-associated membranes (MAMs) serve as a scaffold for the crosstalk between endoplasmic reticulum (ER) and mitochondria, thus playing a pivotal role in the signaling pathways that maintain cellular health. The sigma-1 receptor (Sig-1R) is a ubiquitous ER-resident chaperone localized at MAMs [13] of the central nervous system (CNS). It is involved in the pathogenesis of CNS diseases, especially in neurodegenerative diseases, such as Alzheimer’s Disease (AD) [14], Huntington’s Disease (HD) [15], Parkinson’s Disease (PD) [16], and amyotrophic lateral sclerosis (ALS) [17]. Sig-1R modulates the rate of cell apoptosis. For example, activating Sig-1R reduced ER stress following cerebral ischemic injury by preventing the protein kinase RNA-like ER kinase (PERK) and Inositol-requiring enzyme 1α (IRE1α)-mediated neural apoptosis [18]. Sig-1R also inhibited the production of inflammatory cytokines and reduced mortality by blocking the endonuclease activity of IRE1α in a preclinical rodent model of sepsis [19]. It has been reported previously that a Sig-1R agonist effectively inhibited microglia activation after neural injury [20], but the molecular mechanism of Sig-1R in the context of TBI remains unclear.

Here, we reported results from a study designed to investigate the role of Sig-1R in regulating TBI-induced ER stress and its biological and neurological consequences by analyzing cerebral tissue collected from TBI patients during decompressive craniotomy and by studying mouse models of TBI, including Sig-1R null mice. This study tested the hypothesis that activation of Sig-1R by an exogenous agonist inhibited ER stress-associated microglial activation, apoptosis, and pyroptosis to reduce neuronal cell death and improve cerebrovascular and neurological functions in the mouse model.

## 2. Materials and Methods

### 2.1. Human Brain Tissues

Human tissue was obtained in accordance with an ethically reviewed and approved protocol from Tianjin Medical University General Hospital. Six patients (four males and two females) with severe TBI, defined as post-resuscitation Glasgow coma scale (GCS) scores less than 9, were included. Demographics and clinical characteristics of these patients are shown in Table 1. Control patients were those with cerebrovascular malformation surgery and underwent surgery. None of the patients had any other known neurological disorders.

These patients underwent decompressive craniotomy to remove hematoma and/or to reduce life-threatening high intracranial pressure (case) or surgery to remove cerebral vascular malformation (control). Brain tissues removed during the surgeries were immediately placed in ice-cold Radio-Immunoprecipitation Assay (RIPA) lysis buffer with the protease inhibitor phenylmethanesulfonyl fluoride (PMSF) for 30 min, dispersed, and then centrifuged at 14,000× *g* for 15 min at 4 °C. The supernatant was collected and boiled with sample loading buffer at 99 °C for 10 min.

### 2.2. Experimental Design

**Figure 1:**
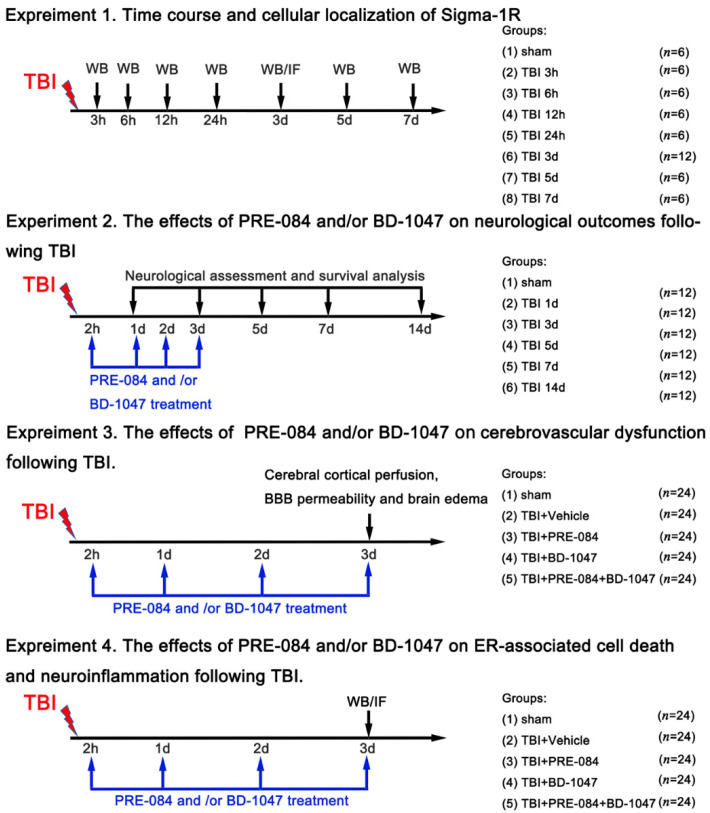
Dosing regimen. Sigma-1R, sigma-1 receptor; TBI, traumatic brain injury; WB, western blot; IF, immunofluorescence; BD-1047, sigma-1 receptor antagonist; PRE-084, sigma-1 receptor agonist; ER, endoplasmic reticulum; BBB, blood-brain barrier.

All mice were randomly assigned to the following. Experiments as described (Figure 1).

Experiment design 1: To examine the expression of the Sig-1R and Caspase-1 in the cortex of mice following TBI, mice were randomly assigned to eight groups: sham groups and TBI groups (3 h, 6 h, 12 h, 24 h, 3 days, 5 days, and 7 days) (*n* = 6 each group). All mice of each group were selected for western blot analyses.

Experiment design 2: To determine the effects of Sig-1R on neurological outcomes following TBI, mice were randomly assigned to five groups: sham group, TBI + vehicle group, TBI + PRE-084 group, TBI + BD-1047 group, and TBI + PRE-084 + BD-1047 group (*n* = 12 each group).

Experiment design 3: To explore the effects of Sig-1R on cerebrovascular dysfunction following TBI, mice were randomly assigned to five groups: sham group, TBI + vehicle group, TBI + PRE-084 group, TBI + BD-1047 group and TBI + PRE-084 + BD-1047 group (*n* = 6 each group). Assessment methods included brain water content, cerebral cortical perfusion, Evans blue extravasation assay, and measurement of lesion volume.

Experiment design 4: To examine the effects of Sig-1R on ER stress-associated cell death and neuroinflammation following TBI, mice were randomly assigned to five groups: sham group, TBI + vehicle group, TBI + PRE-084 group, TBI + BD-1047 group, and TBI + PRE-084 + BD-1047 group (*n* = 6 each group). Assessment methods included western blot, double immunofluorescence staining, measurement of mitochondrial reactive oxygen species content, and Propidium iodide (PI) staining.

### 2.3. Mouse Model of TBI

All experimental procedures were conducted in accordance with the National Institutes of Health Guide for the Care and Use of Laboratory Animals and approved by Tianjin Medical University Animal Care and Use committee. All procedures were approved by the Chinese Small Animal Protection Association Experimental Protocol. Male C57BL/6 mice aged 6–8 weeks and weighing 20–25 g (Experimental Animal Laboratories of the Academy of Military Medical Sciences, Beijing, China) were housed in the temperature (18–22 °C) and humidity (50–60%) controlled vivarium with ad libitum access to food and water under a standard 12-h light/dark cycle.

TBI was induced by a digital, electromagnetically controlled cortical impact (CCI) device (eCCI-6.3 device, Custom Design & Fabrication, Deptford, NJ, USA). Briefly, each mouse was anaesthetized with intraperitoneal administration of 10% chloral hydrate (3 mg/kg) and placed prone in a stereotaxic head frame. After a midline incision over the skull, a 4 mm diameter hole was drilled through the right parietal skull (2.5 mm posterior from the bregma and 2.5 mm lateral to the sagittal suture) to fully expose the dura mater. The mouse was subjected to a unilateral 2.5 mm depth impact at 5 m/s with 200 ms dwell time by a 4-mm-flat impactor tip. The incision was sutured immediately after CCI, and the mouse was placed on a heating pad until recovery from the anesthesia. The sham mice were subjected to the same surgical procedure without infliction of CCI.

The selective Sig-1R agonist PRE-084 (10 mg/kg, Sigma Aldrich, St. Louis, MO, USA) and antagonist BD-1047 (10 mg/kg, Sigma Aldrich) were dissolved individually in 0.9% saline. TBI mice were injected with PRE-084, BD-1047, or 0.9% saline intraperitoneally for three consecutive days beginning at 2 h after TBI. Some TBI mice received BD-1047 first, followed by PRE-084 30 min after BD-1047 administration (Figure 1).

### 2.4. Sig-1R Gene Knockout

We synthesized the recombinant adeno-associated virus serotype 9 (AAV9) carrying clustered regularly interspaced short palindromic repeats-associated 9 (CRISPR/Cas9)-small guide RNA (sgRNA) targeting Sig-R (AAV9-Sig-1R) through a commercial vender (Genechem, Shanghai, China). The target sequence was GCCCAGCCACAACCAGGCGGC. AAV9 that did not contain the RNA insert was used as negative control (NC). Mice were anaesthetized with intraperitoneal administration of 10% chloral hydrate (3 mg/kg) and placed prone in a stereotaxic head frame. After a midline incision over the skull, a small burr hole was drilled through the right parietal skull by a dental drill (0.8 mm posterior, 1.5 mm lateral, and 3.8 mm ventral from the bregma). AAV9-Sig-1R (5 μL, virus titer: 3.34 × 10^12^) or AAV9-NC was injected into the lateral ventricle using a 5 μL Hamilton syringe (Hamilton Company, Reno, NV, USA) at 0.5 μL/min through the hole. After injection, the needle was held for 10 min before retraction and the scalp was sutured. The mice were housed for two weeks to achieve Sig-1R suppression before being subjected to CCI. The Sig-1R knockout mice were also subjected to a unilateral 2.5 mm depth impact at 5 m/s with 200 ms dwell time by a 4-mm flat impactor tip.

### 2.5. Neurological Assessment

Neurological functions were assessed at 1 d, 3 d, 5 d, 7 d, and 14 d after TBI or sham surgery using the well-established modified neurological severity score (mNSS), which consists of motor (muscular state and abnormal action), sensory (visual, tactile), reflex, and balance tests. The mNSS test was graded on a scale of 0–18, in which a score of 18 points indicates maximal neurological deficits and a score of 0 indicates normal function. A lower score indicates better neurological function. Additionally, all subtests of mNSS were repeated twice by two investigators who were blinded to the experimental conditions.

### 2.6. Morris Water Maze

The Morris Water Maze task was used to evaluate the recovery of spatial learning and memory function of the mice as described previously [21]. Briefly, the mice were randomly placed into a quadrant (W, E, S, or N) and the latency to escape to the platform was measured. Each mouse was tested for three trials. The performance was recorded by a video camera and the time spent in the targeting quadrant in 60-s intervals was recorded.

### 2.7. Measurement of Lesion Volume

For quantification of mice brain lesion volume at 14 d after TBI, transverse sections were cut at 120-μm continuous intervals to cover the entire injured cortex as previously described [22]. The slices were then stained with hematoxylin and eosin (H & E, Solarbio, Beijing, China) and imaged under a light microscope (Olympus, Tokyo, Japan). Images were analyzed using National Institute of Health (NIH) ImageJ software (Version 1.4; Bethes, MD, USA). The ipsilateral and contralateral sides were tracked on each slice to obtain the loss of cortical tissue, and this was multiplied by the known distance between slices to obtain the volumes. The volume of cortical lesion was presented as: (contralateral hemisphere volume − ipsilateral hemisphere volume)/contralateral hemisphere volume × 100%.

### 2.8. Brain Water Content

Brain water content (BWC) was calculated at 3 d after TBI by using the wet weight-dry weight method as previously reported [23]. The mice were sacrificed at 3 d after TBI without transcardiac perfusion, and the brains were removed promptly. The brain weight was immediately measured (wet weight) and subsequently placed in an oven at 100 °C for 24 h until a constant weight (dry weight) was measured. The BWC was calculated as: (wet weight − dry weight)/wet weight × 100%.

### 2.9. Cerebral Cortical Perfusion

The cerebral cortical blood perfusion was measured using a Laser speckle imager (PeriCam PSI System, Perimed AB, Jakobsberg, Sweden), as previously described [24]. Briefly, the mice were anesthetized by 10% chloral hydrate injection (3 mg/kg) and positioned prone in a stereotaxic head frame. A midline incision was made over the skull to expose the calvaria, through which the cerebral cortical perfusion was continuously measured for 30 s at the following settings: 10 cm observation height, 2 × 2 cm laser irradiation area, the PSI system at 1386 × 1034 pixels, and the regional spatial contrast was calculated according to the 3 × 3 secondary matrices. To monitor changes to blood perfusion in the region of cortical injury, the mean value of the region of interest was measured (ROI, 15 mm^2^ including lesioned boundary).

### 2.10. Evans Blue Extravasation Assay

The blood-brain barrier (BBB) permeability was evaluated by extravasation of the Evans blue dye (EB) as previous reported [25]. The 2% Evans blue solution (Sigma Aldrich) was administrated intravenously (femoral vein, 4 mL/kg) and circulated in the mice for 2 h as previously reported. Then, the mice were transcardially perfused with phosphate-buffered solution (PBS) to remove the intravascular EB dye, and the brains were collected for raw imaging of EB extravasation. Each cerebral hemisphere was immediately weighed, homogenized in *N*,*N*-dimethylformamide, and incubated at 60 °C for 24 h. The homogenates were centrifuged at 14,000 rpm for 30 min, and absorption of the supernatant was measured by a spectrophotometer at a wavelength of 620 nm. The EB concentration was calculated qualitatively using a standard curve and expressed as micrograms of EB/g of brain tissue using a standardized curve.

### 2.11. Immunofluorescence Staining

For detection of necrotic and dead cells, Propidium Iodide (PI, Sigma Aldrich) was dissolved in saline and administrated intraperitoneally (10 mg/kg) 1 h prior to sacrificing of the mice. The mice were sacrificed at 3 d after TBI and intracardially perfused with ice-cold PBS; the whole brains were removed promptly and post-fixed in 4% paraformaldehyde at 4 °C for 24 h. Then, formaldehyde-fixed tissues were embedded in OCT medium and cut into 8-μm-thick coronal sections using a cryostat (Leica, Model CM1950, Wetzlar, Germany). The frozen sections were incubated overnight at 4 °C with primary antibodies, including NeuN (1:100, Abcam, Cambridge, UK), Iba-1 (1:500, Abcam, UK), GFAP (1:500, Abcam, UK), Caspase-1 (1:200, Santa Cruz Biotechnology, Dallas, TX, USA), Sigma-1R (1:500, Abcam, UK), iNOs (1:500, Cell Signaling Technology, Dallas, TX, USA), Arginase-1 (1:500, Cell Signaling Technology, USA), Claudin-5 (1:500, Invitrogen-Thermo Fisher Scientific, Waltham, MA, USA), and CD31 (1:500, R&D systems, Minneapolis, MN, USA). The sections were then incubated with the appropriate Alexa Fluor-conjugated IgG (1:500, Invitrogen-Thermo Fisher Scientific, USA) for 1 h at 37 °C in the dark. Finally, the sections were covered with 4′,6-diamidino-2-phenylidole (DAPI, Abcam) and imaged by an inverted fluorescence microscope (Olympus, Japan). All results were quantified in lesioned boundaries of six sections per brain at ×200 and/or ×400 magnification using National Institute of Health (NIH) ImageJ software (Version 1.4, Bethesda, MD, USA).

### 2.12. Western Blot Analysis

At 3 h, 6 h, 12 h, 24 h, 3 d, 5 d, and 7 d after TBI, mice were anesthetized with 10% chloral hydrate and intracardially perfused with ice-cold PBS, then ipsilateral cerebral hemispheres were removed promptly. Subsequently, brain samples were homogenized in RIPA and centrifuged at 12,000 rpm at 4 °C for 15 min. The supernatant was collected, and the protein concentration was measured using the Pierce BCA Protein Assay Kit (Thermo Fisher Scientific, USA). Proteins samples and pre-stained molecular weight markers (Thermo Fisher Scientific, USA) were separated by sodium dodecyl sulfate-polyacrylamide gel electrophoresis (SDS-PAGE) and transferred to a PVDF membrane (pore size: 0.45 µm) that was blocked and incubated at 4 °C overnight with the following primary antibodies: Sigma-1R (1:1000, Abcam, UK), Claudin-5 (1:1000, Abcam, UK), GRP78 (1:1000, Abcam, UK), p-PERK (1:1000, Abcam, UK), PERK (1:1000, Abcam, UK), p-IRE1α (1:1000, Abcam, UK), IRE1α (1:1000, Cell Signaling Technology, USA), Bcl-2 (1:500, Cell Signaling Technology, USA), Bax (1:1000, Cell Signaling Technology, USA), CHOP (1:500, Cell Signaling Technology, USA), cleaved-Caspase-3 (1:1000, Cell Signaling Technology, USA), Iba-1(1:500, Abcam, UK), iNOs (1:500, Cell Signaling Technology, USA), Arginase-1(1:500, Cell Signaling Technology, USA), TNF-α (1:500, Cell Signaling Technology, USA), IL-6 (1:500, Cell Signaling Technology, USA), IL-1β (1:500, Cell Signaling Technology, USA), IL-18 (1:500, Cell Signaling Technology, USA), NLRP1 (1:500, Santa Cruz Biotechnology, USA), NLRP3 (1:1000, Abcam, UK), AIM2 (1:1000, Abcam, UK), ASC (1:1000, Abcam, UK), Caspase-1 (1:1000, Abcam, UK), Caspase-1 p20 (1:500, Santa Cruz Biotechnology, USA), Caspase-11 p20 (1:500, Santa Cruz Biotechnology, USA), GSDMD (1:1000, Abcam, UK), β-actin (1:1000, Cell Signaling Technology, USA), and GAPDH (1:1000, Cell Signaling Technology, USA), followed by incubation with appropriate secondary antibodies (1:5000, Cell Signaling Technology, USA) for 1 h at 37 °C. Immunoblots were probed with a Chemiluminescent HRP Substrate (EMD Millipore Corporation, Burlington, VT, USA) and visualized under an imaging system (Bio-Rad, Hercules, CA, USA). Gray value analysis was qualified by Image-J software (Version 1.46r, Wayne Raband, USA). Expression levels of all proteins were normalized against β-actin or GAPDH.

### 2.13. Preparation of Single Cells and Mitochondrial Reactive Oxygen Species Content

The mitochondrial reactive oxygen species (ROS) was stained using the oxidation-sensitive red fluorescence dye Mitosox Red (Thermo Fisher, Molecular Probes: M36008) and measured using flow cytometry. The mice were sacrificed at 3 d after TBI via transcardiac perfusion, and the cerebral hemispheres were removed promptly. The cerebral hemispheres were then incubated with Collagenase IV for 30 min at 37 °C and dispersed constantly by a pipettor during this period. After two washes with PBS, the myelin sheaths of cells were removed using 30% Percoll gradient centrifugation. Finally, the single cells were incubated with 5 µM Mitosox for 10 min at 37 °C, protected from light. The fluorescent intensity of cells was analyzed by flow cytometry.

### 2.14. Terminal Deoxynucleotidyl Transferase dUTP Nick-End Labeling (TUNEL) Assay

For quantification of apoptotic cortical neurons, double staining of NeuN (red) and TUNEL (green) was performed using the In Situ Cell Death Detection kit (Roche, South San Francisco, CA, USA), according to the manufacturer’s instructions. Briefly, frozen sections were counterstained with mouse anti-NeuN (1:100, Abcam) at 4 °C overnight and subsequently incubated with the In Situ Cell Death Detection kit and a secondary donkey anti-mouse Alexa 594 antibody for 1 h at 37 °C in the dark. Finally, the sections were covered with DAPI and visualized under an inverted fluorescence microscope. The number of TUNEL-positive neurons was quantified manually in lesioned boundaries of six sections per brain at ×200 magnification using ImageJ software. Results were expressed as the apoptotic ratio of the total neurons (TUNEL-NeuN double positive stained cells/NeuN stained cells).

### 2.15. Statistical Analysis

Statistical analysis was performed with Graph-Pad Prism software (Graph Pad Software, Version8.1.2 San Diego, CA, USA). All data were analyzed using Student’s *t*-test (two groups) or one-way analysis of variance (ANOVA) followed by Tukey’s multiple comparison post hoc test (more than two groups). All data are expressed as means ± standard error of the mean (SEM). A probability value of *p* < 0.05 was considered statistically significant.

## 3. Results

### 3.1. TBI Upregulated the Expression of Sig-1R and GRP78 in TBI Patients and Mice Subjected to TBI

**Figure 2:**
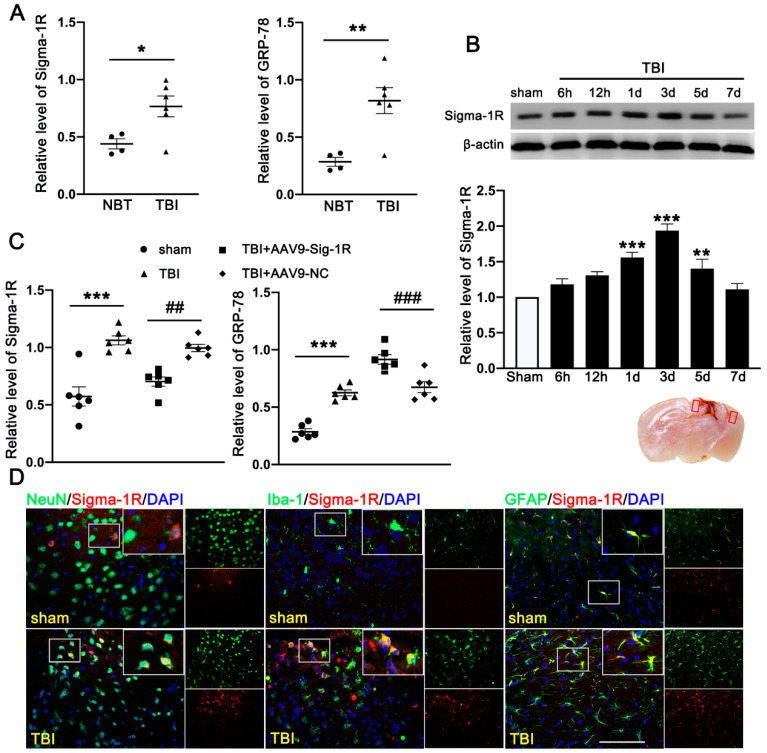
Time course expression and cellular localization of endogenous Sig-1R in mice subjected to TBI. (**A**) Quantitative analysis of the relative protein expression of Sig-1R and GRP78 in cerebral tissues from TBI (*n* = 6) and AVM patients (NBTs) (*n* = 4). (**B**) The time-dependent expression of Sig-1R in the ipsilateral cerebral hemispheres after TBI. *n* = 6 per group. (**C**) Quantitative analysis of the expression of Sig-1R and GRP78 in the ipsilateral cerebral hemispheres from TBI mice indicated that knocking out the expression of Sig-1R promoted increase of GRP78 expression after TBI. *n* = 6 per group. (**D**) Double immunofluorescence staining of Sig-1R (red) with neurons (NeuN, green), microglia (Iba-1, green), astrocytes (GFAP, green) showed that Sig-1R was primarily localized in astrocytes in the sham groups, whereas Sig-1R was abundantly expressed in neurons, microglia, and astrocytes in the ipsilateral peri-lesion cortex after TBI. Nuclei were stained with DAPI (blue). Two small red squares within the coronal section of the brain indicated the areas where the microphotographs were taken. *n* = 6 per group Scale bar = 100 μm. Data are represented as mean ± SEM. * *p* < 0.05, ** *p* < 0.01 and *** *p* < 0.001 vs. sham group; ## *p* < 0.01 and ### *p* < 0.001 vs. TBI +AAV9-NC group; one-way ANOVA, Tukey’s post hoc test.

The clinical study comprised of six TBI patients and four cerebrovascular malformation patients. We detected significantly more Sig-1R and the UPR marker (GRP78) in the brain homogenates from TBI patients than those from AVM (0.401 ± 0.1177) (Figure 2A). Consistent with the patient study, the expression of Sig-1R in the ipsilateral cerebral hemispheres was increased in a time-dependent manner when compared with sham mice, reaching the peak level at 3d after TBI followed by decreasing to the level comparable to that of sham mice at day 7 post-surgery (Figure 2B). Sig-1R expression was significantly reduced in TBI mice infected with AAV9-Sig-1R (−0.293 ± 0.0751), but not changed in the TBI mice infected with the control vector (Figure 2C), suggesting that the Sig-1R gene was suppressed. The expression of GRP78 was significantly increased in the cerebral tissue from Sig-1R knockout mice 3 days after TBI as compared with control mice (AAV-9-NC) (0.425 ± 0.0515) (Figure 2C). Immunofluorescence staining showed that Sig-1R was primarily localized in astrocytes (GFAP) of the sham mice. In contrast, Sig-1R was not only abundantly expressed in astrocytes, but also in neurons (NeuN) and microglia (Iba-1) of TBI mice (Figure 2D).

### 3.2. Activation of Sig-1R Improved Neurological Outcomes and Cerebrovascular Function after TBI

**Figure 3:**
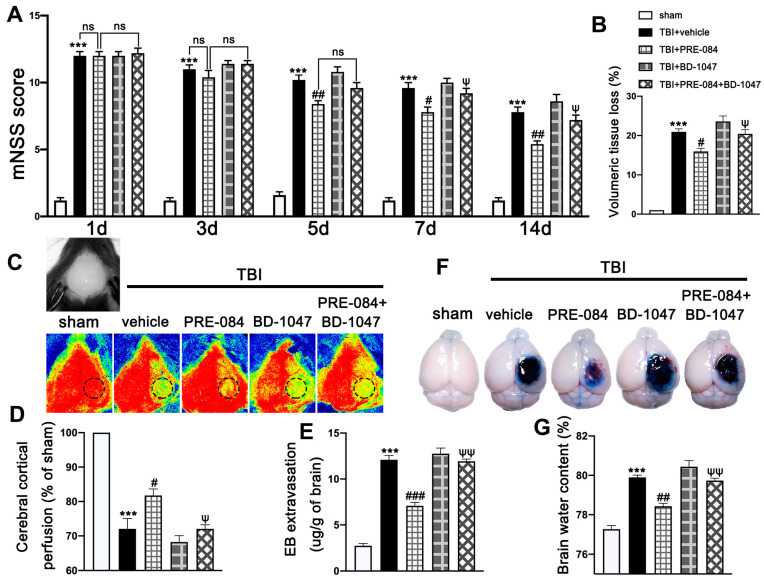
The effects of Sigma-1 receptor (Sig-1R) agonist PRE-084 and antagonist BD-1047 on neurological outcomes and cerebrovascular function post-injury. (**A**) Neurological function score of mice were assessed using modified neurological severity score (mNSS) at 1 d, 3 d, 5 d, 7 d, and 14 d after TBI. *n* = 12 per group. (**B**) Quantitative analysis of lesion volume at 14 d post-injury. *n* = 6 per group. (**C**) Representative cerebral cortical perfusion images and (**D**) quantitative analysis of cerebral cortical perfusion in cortical injury area (small black and dotted circle) after TBI. *n* = 6 per group. (**E**) Quantitative analysis of brain water content of mice in different groups at 3 d after TBI. *n* = 6 per group. (**F**) Representative images of Evans blue (EB) leakage and (**G**) quantitative analysis of EB extravasation at 3 d after TBI. *n* = 6 per group. Data are represented as mean ± SEM. *** *p* < 0.001 vs. sham group; # *p* < 0.05, ## *p* < 0.01 and ### *p* < 0.001 vs. TBI + Vehicle group; ψ *p* < 0.05, ψψ *p* < 0.01 vs. TBI + PRE-084 group; ns, no significance; one-way ANOVA, Tukey’s post hoc test.

Neurological function defined by mNSS was progressively and significantly impaired in TBI mice, being the worst on day 1 post-TBI and then gradually improving (Figure 3A). In contrast, mNSS was significantly improved in TBI mice receiving PRE-084, especially 5 days after TBI, as compared with those receiving the vehicle buffer (Figure 3A). The neuroprotective effects of PRE-084 were again reversed by BD-1047 from day 7 after TBI (Figure 3A). Next, we evaluated the performance improvement in PRE-084 treated mice. PRE-084 treated mice moved to the platform with a shorter latency when compared to vehicle or BD-1047 treated mice (Supplementary Figure S1A). Similarly, PRE-084 treated mice exhibited a significant performance for the correct quadrant and spent more time in correct quadrant (Supplementary Figure S1B). The effects of PRE-084 were blocked by the Sig-1R antagonist BD-1047 (Supplementary Figure S1B). PRE-084-treated TBI mice significantly reduced the volume of cerebral lesion at 14 d after TBI (−4.975 ± 1.334), as compared with TBI mice receiving the vehicle buffer, whereas BD-1047 reversed the protective effect of PRE-084 (4.475 ± 1.334) (Figure 3B).

The cerebral cortical perfusion surrounding the injury area was significantly reduced at 3 d after TBI compared with that of sham mice (−29.131 ± 2.699) (Figure 3C,D). Treatment with PRE-084 significantly increased the cerebral cortical perfusion when compare with the vehicle treatment. (11.600 ± 2.699). The Sig-1R antagonist BD-1047 given alone further reduced cerebral perfusion of TBI mice and also reversed the protective effect of PRE-084 (−10.370 ± 2.699) (Figure 3C,D). Similarly, cerebral water content in vehicle-treated mice was significantly higher than those treated with PRE-084 (1.467 ± 0.2176) (Figure 3E). Finally, BBB permeability qualified by Evans blue extravasation at 3 d after TBI was significantly reduced in PRE-84-treated mice (−5.025 ± 0.5812), but not in those receiving PRE-084 together with BD-1047 (Figure 3F,G).

**Figure 4:**
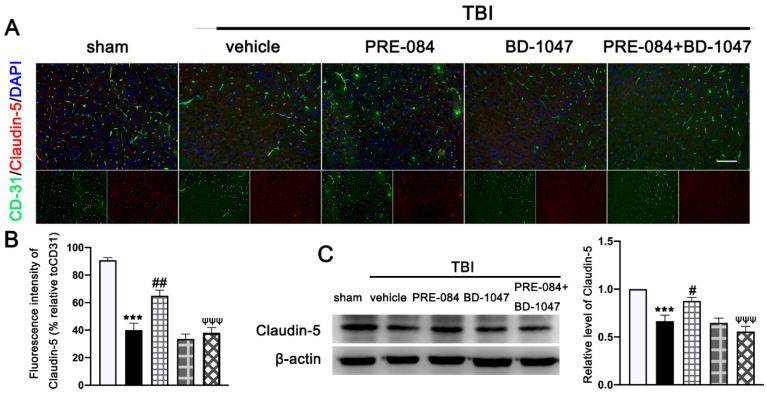
The effects of Sigma-1 receptor (Sig-1R) agonist PRE-084 and antagonist BD-1047 on blood-brain barrier (BBB) permeability after traumatic brain injury (TBI). (**A**) Representative double immunofluorescence staining of tight junction protein Caludin-5 (red) with platelet endothelial cell adhesion molecule 1 (CD-31) (green) and (**B**) quantitative fluorescence intensity analysis of Caludin-5 (relative to CD31) in the ipsilateral peri-lesion cortex after TBI. Nuclei were stained with DAPI (blue). *n* = 6 per group. Scale bar = 100 μm. (**C**) Representative Western blot band and quantitative analysis of the relative expression of Claudin-5 at 3 d after TBI. *n* = 6 per group. Scale bar = 100 μm. Data are represented as mean ± SEM. *** *p* < 0.001 vs. sham group; # *p* < 0.05, ## *p* < 0.01 vs. TBI + vehicle group; ψψψ *p* < 0.001 vs. TBI + PRE-084 group; one-way ANOVA, Tukey’s post hoc test.

Double immunofluorescence staining showed that TBI mice receiving the vehicle had less Claudin-5-positive vessels (labeled by the endothelial marker CD31) in lesioned boundary than sham mice at 3 d post-injury (−50.650 ± 5.762) (Figure 4A,B). PRE-084 restored the Claudin 5 expression (24.940 ± 5.762) and its effect was blocked by BD-1047 (−26.920 ± 5.762) (Figure 4A,B). This observation was further supported by the Western blots, showing that expression of Claudin-5 after TBI was significantly reduced (−0.334 ± 0.0647) (Figure 4C), which was rescued in mice receiving PRE-084 (0.210 ± 0.0647) and suppressed again in mice treated with PRE-084 and BD-1047(−0.319 ± 0.0647).

### 3.3. PRE-084 Suppressed Mitochondrial Dysfunction and ER Stress-Mediated Neuronal Apoptosis after TBI

**Figure 5:**
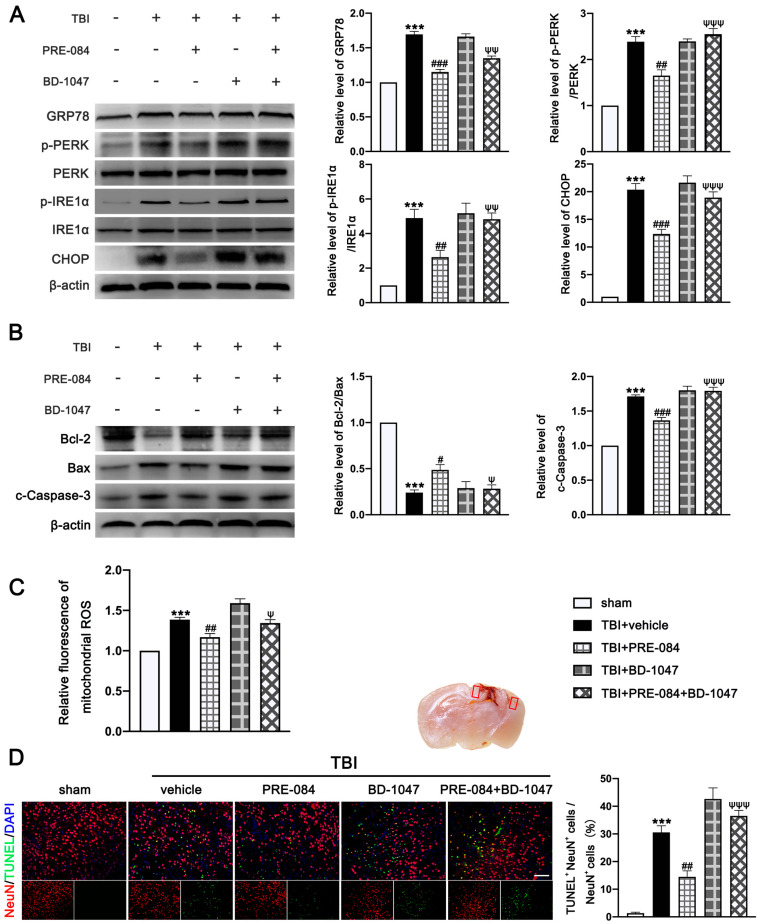
The effects of Sigma-1 receptor (Sig-1R) agonist PRE-084 and antagonist BD-1047 on endoplasmic reticulum (ER) stress-mediated neuronal apoptosis and mitochondrial dysfunction after TBI. (**A**) Representative Western blot bands and quantitative analyses of the relative expressions of GRP78, phosphorylation of protein kinase RNA-like ER kinase (p-PERK)/PERK, phosphorylation of inositol-requiring enzyme 1α (p-IRE1α)/IRE1α and C/EBP-homologous protein (CHOP) in the ipsilateral cerebral hemispheres after TBI. *n* = 6 per group. (**B**) Representative Western blot bands and quantitative analyses of the relative expressions of B-cell lymphoma-2 (Bcl-2)/Bcl-2-associated X (Bax) and cleaved-Caspase-3 after TBI. *n* = 6 per group. (**C**) Quantitative analyses of relative fluorescence of mitochondrial reactive oxygen species (ROS). (**D**) Double immunofluorescence staining of Terminal deoxynucleotidyl transferase dUTP nick-end labeling (TUNEL) (green) with neurons (NeuN, red) and quantitative analysis of TUNEL+ NeuN+ cells/NeuN+ cells in the lesioned boundary after TBI. Nuclei were stained with DAPI (blue). *n* = 6 per group. Scale bar = 100 μm. Data are represented as mean ± SEM. *** *p* < 0.001 vs. sham group; # *p* < 0.05, ## *p* < 0.01 and ### *p* < 0.001 vs. TBI + Vehicle group; ψ *p* < 0.05, ψψ *p* < 0.01, ψψψ *p* < 0.001 vs. TBI + PRE-084 group; one-way ANOVA, Tukey’s post hoc test.

The protein expression of GRP-78 (0.693 ± 0.0431), p-PERK/PERK (1.387 ± 0.1366), p-IRE1α/IRE1α (3.900 ± 0.591), and C/EBP-homologous protein (CHOP) (19.370 ± 1.335) were significantly increased at 3 d post-TBI (Figure 5A). PRE-084 prevented the increased expression of these factors and the inhibitory effects of PRE-084 were abolished by BD-1047 (Figure 5A). We also detected significant a decrease in the expression of B-cell lymphoma-2 (Bcl-2)/Bcl-2-associated X (Bax) (−0.760 ± 0.058) and an increase in cleaved-Caspase-3 (0.7133 ± 0.0506) in ipsilateral cerebral hemispheres of TBI mice at 3 d post-injury (Figure 5B), indicating severe apoptosis and the production of mitochondrial ROS. Both processes were reduced in mice receiving PRE-084 after TBI when compared with TBI mice receiving the vehicle buffer (Figure 5B). The effects of PRE-084 were blocked by the Sig-1R antagonist BD-1047 (Figure 5B). Consistent with findings from immunoblots, flow cytometry analysis revealed that the mitochondrial ROS was markedly reduced in the ipsilateral cerebral hemispheres of TBI mice treated with PRE-084 as compared with those with the vehicle buffer (−0.215 ± 0.060), whereas BD-1047 treatment reversed the effect of PRE-084 (0.175 ± 0.060) (Figure 5C). Finally, TUNEL staining detected less TUNEL-positive neurons in the lesioned boundary of TBI mice receiving PRE-084 at 3 d post TBI (−16.133 ± 3.500) (Figure 5D). This anti-apoptosis effect of PRE-084 was abolished in mice receiving PRE-84 and BD-1047 together (−22.075 ± 3.500).

### 3.4. PRE-084 Attenuated Inflammasome-Mediated Pyroptosis

**Figure 6:**
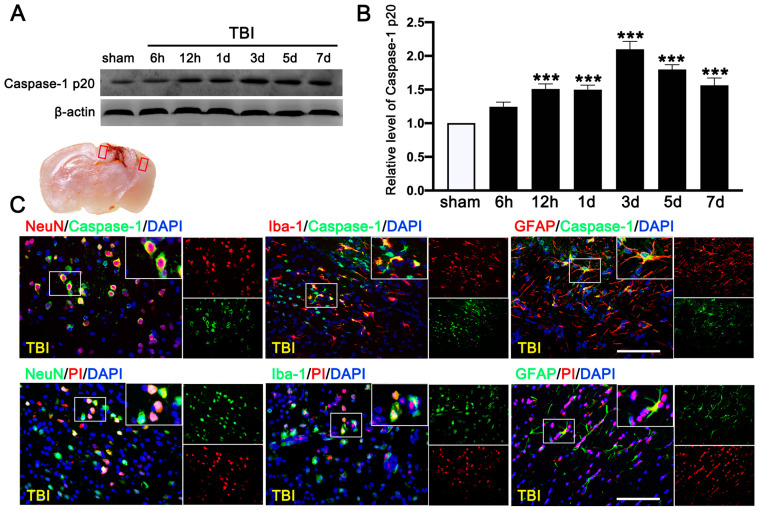
Time course and cellular location of Caspase-1-mediated pyroptosis after traumatic brain injury (TBI). (**A**) Representative Western blot and (**B**) quantitative analysis of the time-dependent expression of Caspase-1 p20 in the ipsilateral cerebral hemispheres after TBI. *n* = 6 per group. (**C**) Double immunofluorescence staining of Caspase-1 (green) with neurons (NeuN, red), microglia (Iba-1, red), astrocytes (GFAP, red) in the lesioned boundary of cortex after TBI and staining of propidium lodide (PI) (red) with neurons (NeuN, green), microglia (Iba-1, green), and astrocytes (GFAP, green) in the lesioned boundary of cortex after TBI. Nuclei were stained with DAPI (blue). *n* = 6 per group. Scale bar = 100 μm. Data are represented as mean ± SEM. *** *p* < 0.001 vs. sham group; one-way ANOVA, Tukey’s post hoc test.

We first evaluated the expression of cleaved-caspase-1 p20 in the ipsilateral cerebral hemispheres by Western blot at 0 h (sham), 6 h, 12 h, 1 d, 3 d, 5 d, and 7 d after TBI. We found that c-caspase-1 p20 increased its expression in a time-dependent manner and peaked at 3 d after TBI mice (Figure 6A,B), followed by a gradual decrease, but it remained higher than that in sham mice at 7 d after TBI. Double immunofluorescence staining detected caspase-1-mediated pyroptosis in neurons (NeuN^+^), microglia (Iba-1^+^), and astrocytes (GFAP^+^) in the lesioned boundary at 3 d after TBI (Figure 6C). The necrotic and dead cells were visualized by PI staining at 3 d after TBI. PI-positive cell death was detected in neurons (NeuN), microglia (Iba-1), and astrocytes (GFAP) (Figure 6C), consistent with the result of cellular distribution of c-caspase-1 p20.

**Figure 7:**
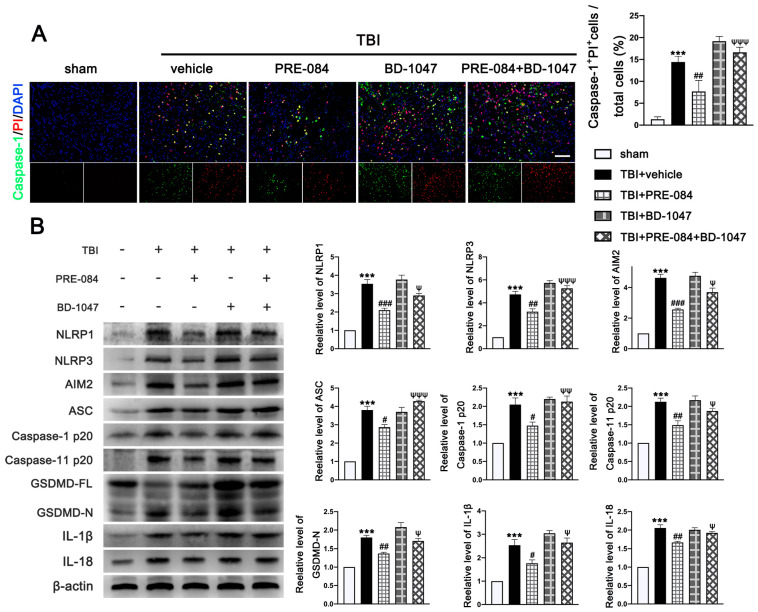
The effects of Sigma-1 receptor (Sig-1R) agonist PRE-084 and antagonist BD-1047 on inflammasome-mediated pyroptosis after traumatic brain injury (TBI). (**A**) Representative double immunofluorescence staining of Caspase-1 (green) with propidium lodide (PI) (red) in the lesioned boundary of cortex after TBI. Nuclei were stained with DAPI (blue) and quantitative analysis of Caspase-1+ PI+ cells/total cells in the lesioned boundary of cortex after TBI. *n* = 6 per group. Scale bar = 100 μm. (**B**) Representative western blot bands of NLR family, pyrin domain-containing 1 (NLRP1), NLR family, pyrin domain-containing 3 (NLRP3), absent in melanoma-2 (AIM2), adaptor protein apoptosis-associated speck-like protein-containing a caspase recruitment domain (ASC), Pro-Caspase-1, Caspase-1 p20, full-length gasdermin D (GSDMD-FL), amino terminal-domain gasdermin D (GSDMD-N), interleukin 1β (IL-1β), and interleukin 18 (IL-18) as well as quantitative analyses of the relative expression of proteins NLRP1, NLRP3, AIM2, ASC, Caspase-1 p20, GSDMD-N, IL-1β, and IL-18 in the ipsilateral cerebral hemispheres after TBI. *n* = 6 per group. Data are represented as mean ± SEM. *** *p* < 0.001 vs. sham group; # *p* < 0.05, ## *p* < 0.01 and ### *p* < 0.001 vs. TBI + vehicle group; ψ *p* < 0.05, ψψ *p* < 0.01, ψψψ *p* < 0.001 vs. TBI + PRE-084 group; one-way ANOVA, Tukey’s post hoc test.

The PRE-084 treatment significantly reduced the percent of Caspase-1^+^/PI^+^ cells and this anti-pyroptosis effect of PRE-084 was abolished in mice also receiving BD-1047 (Figure 7A). Moreover, the inflammasome-associated protein expression of the NLR family, pyrin domain-containing 1 (NLRP1) (2.533 ± 0.2357), NLR family, pyrin domain-containing 3 (NLRP3) (3.733 ± 0.3018), absent in melanoma-2 (AIM2) (3.633 ± 0.2757), adaptor protein apoptosis-associated speck-like protein-containing a caspase recruitment domain (ASC) (2.812 ± 0.2268), Caspase-1 p20 (1.050 ± 0.1651), Caspase-11 p20 (1.127 ± 0.1136), amino terminal-domain gasdermin D (GSDMD-N) (0.7997 ± 0.08744), interleukin 1β (IL-1β) (1.533 ± 0.2323), and interleukin 18 (IL-18) (1.057 ± 0.0726) were significantly increased post-TBI (Figure 7B). The upregulation of these proinflammatory markers was blocked by PRE-084 and enhanced by BD-1047 (Figure 7B). BD-1048 also reversed the the PRE-084 effect.

### 3.5. PRE-084 Promoted Microglia/Macrophages Activation and Inhibited Release of Inflammatory Cytokines following TBI

**Figure 8:**
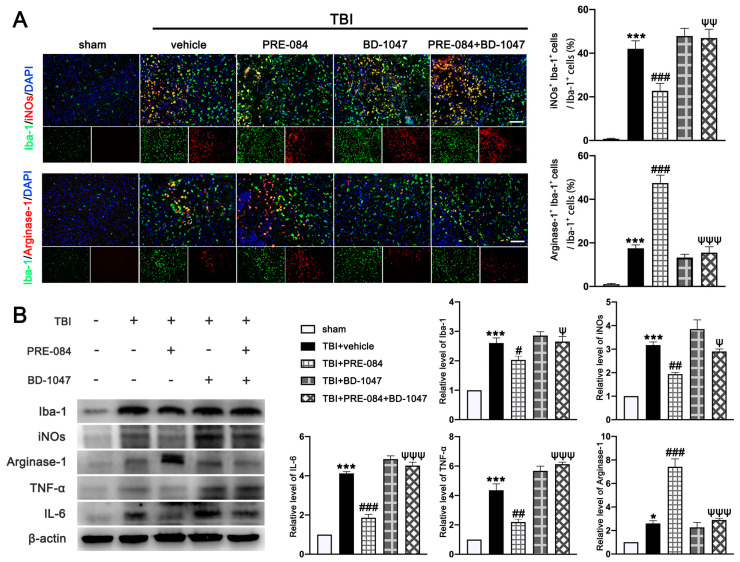
The effects of Sigma-1 receptor (Sig-1R) agonist PRE-084 and antagonist BD-1047 on regulating microglia/macrophages activation after traumatic brain injury (TBI). (**A**) Representative double immunofluorescence staining of microglia (Iba-1, green) with a potential pro-inflammatory phenotype marker inducible nitric oxide synthase (iNOs) (red) and a potential anti-inflammatory phenotype (M2) marker Arginase-1 (red) in the lesioned boundary of cortex after TBI and quantitative analysis of iNOs+ Iba-1+ cells/Iba-1+ cells and Arginase-1+ Iba-1+ cells/Iba-1+ cells in the lesioned boundary of cortex after TBI. (**B**) Representative Western blot bands and quantitative analyses of Iba-1, iNOs, Arginase-1, tumor necrosis factor α (TNF-α), and interleukin 6 (IL-6) in the ipsilateral hemispheres at 3 d after TBI. Data are represented as mean ± SEM. * *p* < 0.05 and *** *p* < 0.001 vs. sham group; # *p* < 0.05, ## *p* < 0.01 and ### *p* < 0.001 vs. TBI+ vehicle group; ψ *p* < 0.05, ψψ *p* < 0.01, ψψψ *p* < 0.001 vs. TBI + PRE-084 group; one-way ANOVA, Tukey’s post hoc test.

It has been well established that Sig-1R expressed at MAMs is involved in the activation of microglia in stroke [26], Parkinson’s disease [16], and Amyotrophic Lateral Sclerosis [27]. We found that the activated microglia/macrophages labelled with inducible nitric oxide synthase (iNOs) (a potential pro-inflammatory mediator) (40.750 ± 1.945) were significantly increased after TBI (Figure 8A). The microglial/macrophages activation was not significantly reduced in TBI mice receiving PRE-084 (Figure 8A). In contrast, the activated microglia stained with Arginase-1 (a potential anti-inflammatory mediator) were increased after TBI (17.450 ± 1.640) and remarkably accelerated by PRE-084 treatment (25.41 ± 1.640) (Figure 8A). This observation was further validated by immunoblotting of cerebral tissue homogenates (Figure 8B). More importantly, the Sig-1R antagonist BD-1047 also reversed this effect of PRE-084 (Figure 8B). Consistent with the microglial/macrophage cells undergoing activation, ipsilateral cerebral cells from TBI mice secreted significantly more pro-inflammatory mediators, tumor necrosis factor α (TNF-α) (3.357 ± 0.3739), and interleukin 6 (IL-6) (3.120 ± 1.962) than sham mice measured at 3 d post-TBI (Figure 8B). The development of this pro-inflammatory phenotype was prevented in TBI mice receiving PRE-084, the effect of which was blocked in mice that also received BD-1047 (Figure 8B).

## 4. Discussion

**Figure 9:**
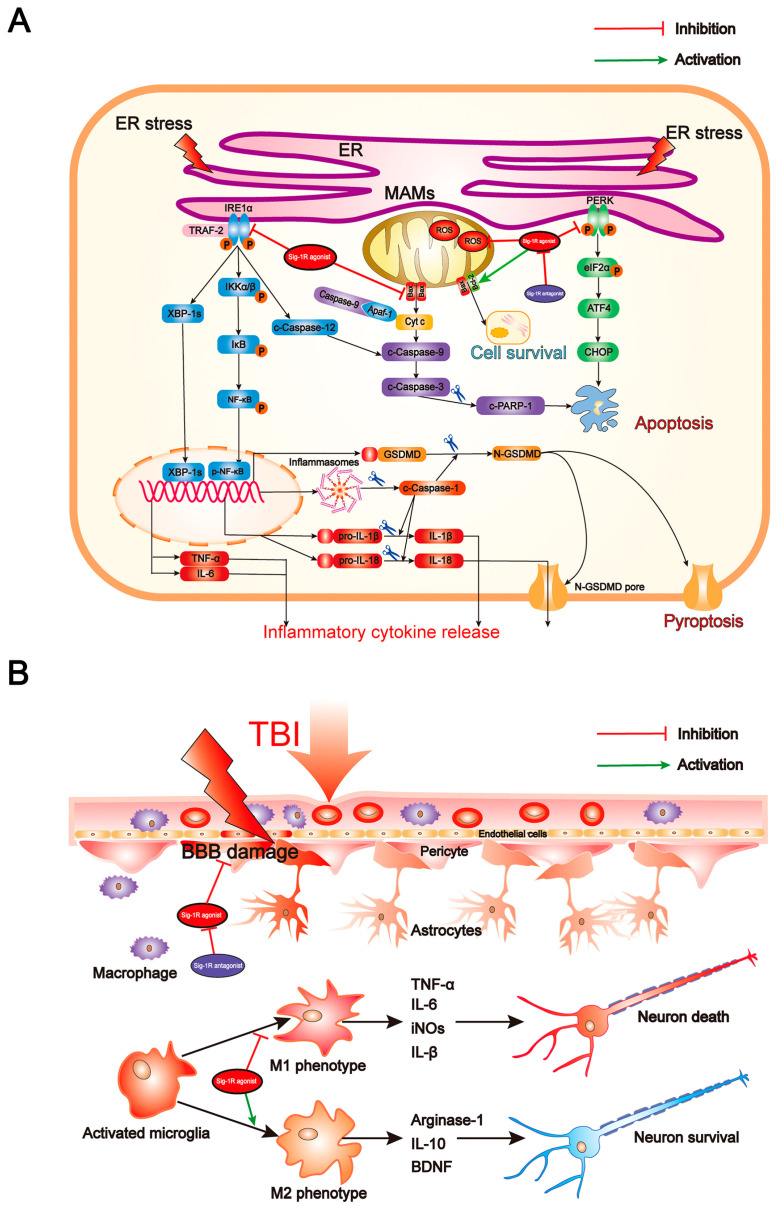
Summary of the underlying mechanisms of the protective effect of Sigma-1 receptor against endoplasmic reticulum stress-mediated apoptosis, pyoptosis, and inflammatory response in mice subjected to traumatic brain injury. (**A**) Sigma-1 receptor agonist inhibits endoplasmic reticulum stress-mediated and mitochondria-mediated apoptotic, pyroptotic and inflammatory pathways activation. (**B**) Sigma-1 receptor agonist confer neuroprotection via amelioration of blood-brain barrier damage, microglial activation and polarization, and neuronal death after TBI.

In the current study, we first demonstrated that Sig-R and GRP-78 expression were significantly increased in TBI patients’ brain tissues, indicating that ER stress in the brain after TBI was evoked and meanwhile neural expression of Sig-1R as a part of the home-defense mechanism was up-regulated. Consistently, we detected the same results in the brain of mice subjected to TBI. As previous studies described that Sig-R plays an important role in neuroprotection [20] but the molecular mechanism of Sig-1R in the context of TBI remains unclear. We then investigated the effects of Sig-1R on secondary brain injury following TBI and whether the mechanism by which Sig-1R exerts such strong neuroprotective was through inhibiting ER stress. First, we found that knockout of Sig-R by CRISPR/CAS9 in mice remarkably aggravated ER-stress after TBI. Then, we also detected that activation of Sig-1R with PRE-084 significantly alleviated the ER stress-induced cell death and microglia activation, and rescued the lesion volume, neurological deficits, and cerebrovascular dysfunction after TBI (Figure 9A,B). Conversely, these neuroprotective effects of PRE-084 were mostly blocked in the presence of Sig-1R antagonist BD-1047. All these results together indicate that activation of Sig-1R may provide strong neuroprotective and anti-inflammatory effects by inhibiting ER stress following TBI.

Secondary cell death and neuroinflammation are two major pathological hallmarks of TBI and are associated with cerebrovascular dysfunction and neurological deficits post-injury. Extensive findings have elucidated that persistent and devastating ER stress is involved in neuronal apoptosis after TBI and inhibition of ER stress significantly reduced neuronal apoptosis and improved neurological function post-injury [11]. Growing evidence has elucidated that, after TBI, inflammasome-mediated pyroptosis plays a critical role in determining cell fate and regulating immune response [5,28] and ER stress plays important roles in regulating inflammasome-mediated pyroptosis [29,30,31]. Neuroinflammation orchestrated by activated microglia plays an extremely critical role in aggravating secondary brain injury and deterring brain repair after TBI. Recently, it has been broadly demonstrated that ER stress plays a critical role in modulating microglia polarization and neuroinflammation, in which inhibition of ER stress, especially IRE1α and PERK-associated downstream signaling pathways, efficiently attenuates microglia-mediated neuroinflammation. As previous studies have identified that microglia/macrophages not only divide to M1 and M2 phenotypes, but also include Mhem, MHb, Mox, and M4 phenotypes [32,33,34], it is complicated to explore microglial activation phenotypes transformation during TBI in vivo. In the present study, we only detected that Sig-1R effectively inhibited microglia-mediated potential pro-inflammatory mediators release (iNOs, TNF-α, IL-6), but promoted microglia-mediated potential anti-inflammatory mediator expression (Arginase-1). Thus, the approach targeting the excessive ER stress-mediated cell death (apoptosis and pyroptosis) and microglia-mediated neuroinflammation could provide a promising therapeutic strategy for TBI.

Sig-1R is constitutively engaged in the modulation of UPR, in which Sig-1R directly or indirectly regulates three ER sensors and their downstream signaling pathways (Figure 9A). Previous studies have confirmed that, in response to ER stress, Sig-1R is mainly involved in regulating both UPR branches (PERK-mediated and IRE1α-mediated branches). Although numerous studies have revealed that activating Sig-1R efficiently suppressed apoptosis via the PERK/CHOP pathway [13,18,35,36,37], our present study is the first to document that activation of Sig-1R with the selective agonist, PRE-084, in mice subjected to TBI significantly decreases PERK/CHOP pathway-related neuronal apoptosis. In addition, in a previous in vitro study using immunoprecipitation assay, it was found that, upon ER stress, Sig-1R directly interacted with IRE1α and regulated its dimerization and phosphorylation [38]. It has been confirmed that Sig-1R activation efficiently reduced the expression of p-IRE1α in response to severe ER stress following cerebral ischemia injury [18,36]. Collectively, these observations first unraveled the underlying mechanism by which Sig-1R activation may modulate ER stress-associated apoptosis, pyroptosis, and microglia-mediated neuroinflammation in a mouse model of TBI.

Several limitations of the present study need to be acknowledged. First, since the clinical data of TBI patients were mostly patients with polytrauma, even if we obtained the insult brain for research, it is not enough to prove that the up-regulation of ER stress responses is simply due to brain trauma, which may be due to other system/organ injuries. Second, since the double immunofluorescence staining showed that Sig-1R was extensively expressed in neurons, microglia, and astrocytes, further investigations are necessary to reveal the roles of Sig-1R in astrocytes after TBI. In addition, we did not specifically demonstrate the mechanisms by which Sig-1R modulates IRE1α and PERK. Although the transient interaction between Sig-1R and IRE1α has been elucidated in a previous study, the dynamical interaction was not unraveled with the administration of diverse Sig-1R ligands. Similarly, the interaction between Sig-1R and PERK remains poorly understood. Thus, further studies using co-immunoprecipitation (Co-IP) and/or other assays are needed to determine the detailed interactions between Sig-1R, IRE1α, and PERK. Last but not least, in this study, the mechanism by which Sig-1R exerts such profound anti-inflammatory and neuroprotective activities was not demonstrated. Further studies are needed to explore other Sig-1R-mediated signaling pathways that modulate cell death and immune responses in the context of brain insult.

## 5. Conclusions

In conclusion, the present study first demonstrated that GRP-78 expression was significantly increased in both TBI patients brain samples and TBI mice brain samples, indicating that ER stress in brain after TBI was evoked. Then, we detected that the neural expression of Sig-1R as a part of the home-defense mechanism was up-regulated in brain tissues from both TBI patients and TBI mice. Then, we found that activation of Sig-1R with PRE-084 attenuated ER stress-associated apoptosis, pyroptosis, and neuroinflammation, as well as restored cerebrovascular function and neurological function in mice subjected to TBI. Thus, Sig-1R activation may provide a promising strategy for translating into clinical therapeutic approaches targeting pharmacological treatment in patients with TBI.

## Figures and Tables

**Table 1:** Demographics and clinical characteristics of human brain tissues from acute traumatic brain injury and arteriovenous malformation.

Case	Age	Gender	Cause of Injury	Other Injuries	Time Post- Injury (h)	Region of Surgery	GCS
Traumatic brain injury	40	Male	Falling injury	None	20	Left temporal lobe	6
Traumatic brain injury	62	Male	Traffic accident	None	16	Right temporal lobe	8
Traumatic brain injury	37	Female	Traffic accident	None	18	Right temporal lobe	6
Traumatic brain injury	75	Male	Traffic accident	None	25	Right parietal lobe	5
Traumatic brain injury	59	Male	Traffic accident	None	29	Left frontal lobe	8
Traumatic brain injury	66	Female	Struck by object	None	22	Left parietal lobe	7
Arteriovenous malformation	48	Male	-	None	-	Right temporal lobe	-
Arteriovenous malformation	33	Female	-	None	-	Right parietal lobe	-
Arteriovenous malformation	42	Female	-	None	-	Left temporal lobe	-
Arteriovenous malformation	38	Male	-	None	-	Right frontal lobe	-

## Data Availability

The datasets supporting the conclusions of this article are included within the article. All materials used in this manuscript will be made available to researchers and are subject to confidentiality.

## Supplementary Materials

The following supporting information can be downloaded at: https://www.mdpi.com/article/10.3390/jcm11092348/s1, Figure S1. The effects of Sigma-1 receptor (Sig-1R) agonist PRE-084 and antagonist BD-1047 on behavioral effects after traumatic brain injury (TBI).

## Abbreviations

TBI	Traumatic brain injury
ER	Endoplasmic reticulum
UPR	Unfolded protein response
Sig-1R	Sigma-1 receptor
MAMs	Mitochondria-associated membranes
IRE1α	Inositol-requiring enzyme 1α
GRP78	78 kDa glucose-regulated protein
CNS	Central nervous system
AD	Alzheimer’s Disease
HD	Huntington’s Disease
PD	Parkinson’s Disease
ALS	Amyotrophic lateral sclerosis
IRE1α	Inositol-requiring enzyme 1α
AAV9	Adeno-associated virus serotype 9
mNSS	Modified neurological severity score
CCI	Controlled cortical impact
