# Supplementary material for: Activation of Sigma-1 Receptor Alleviates ER-Associated Cell Death and Microglia Activation in Traumatically Injured Mice
Source: J Clin Med. 2022 Apr 22;11(9):2348. doi: 10.3390/jcm11092348 (PMC9102000; doi:10.3390/jcm11092348)
Supplement: Supplementary file 1 [file jcm-11-02348-s001.zip › jcm-1619472-supplementary.pdf]

Journal of
Clinical Medicine

MDPI

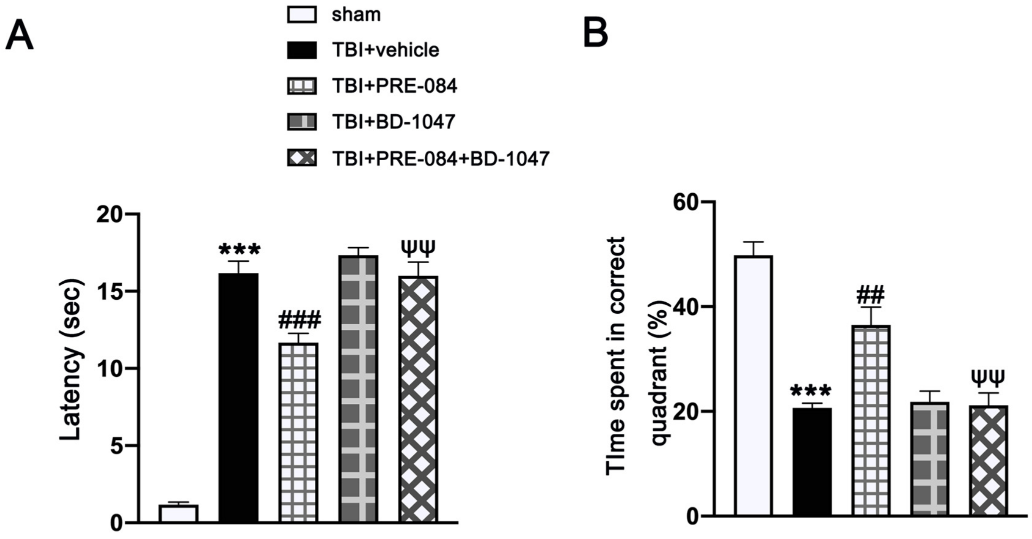

Figure S1. The effects of Sigma-1 receptor (Sig-1R) agonist PRE-084 and antagonist BD-1047 on behavioral effects after traumatic brain injury (TBI). (**A**) Quantitative analysis of latency and (**B**) the time spent in the correct quadrant in different groups after TBI. $n = 6$ per group. Data are represented as mean ± SEM. *** $p < 0.001$ vs. sham group; ## $p < 0.01$ and ### $p < 0.001$ vs. TBI + Vehicle group; ψψ $p < 0.01$ vs. TBI + PRE-084 group; one-way ANOVA, Tukey's post hoc test.
